# Biomimetic Soft Underwater Robot Inspired by the Red Muscle and Tendon Structure of Fish

**DOI:** 10.3390/biomimetics8020133

**Published:** 2023-03-24

**Authors:** Daisuke Aragaki, Toi Nishimura, Ryuki Sato, Aiguo Ming

**Affiliations:** Department of Mechanical Engineering and Intelligent Systems, The University of Electro-Communications, Tokyo 182-8585, Japan

**Keywords:** bio-inspired robot, fish robot, soft robotics, carangiform swimming mode, muscle and tendon structure, multi-links, shape memory alloy, undulation motion

## Abstract

Underwater robots are becoming increasingly important in various fields. Fish robots are attracting attention as an alternative to the screw-type robots currently in use. We developed a compact robot with a high swimming performance by mimicking the anatomical structure of fish. In this paper, we focus on the red muscles, tendons, and vertebrae used for steady swimming of fish. A robot was fabricated by replacing the red muscle structure with shape memory alloy wires and rigid body links. In our previous work, undulation motions with various phase differences and backward quadratically increasing inter-vertebral bending angles were confirmed in the air, while the swimming performance in insulating fluid was poor. To improve the swimming performance, an improved robot was designed that mimics the muscle contractions of mackerel using a pulley mechanism, with the robot named UEC Mackerel. In swimming experiments using the improved robot, a maximum swimming speed of 25.8 mm/s (0.11 BL/s) was recorded, which is comparable to that of other soft-swimming robots. In addition, the cost of transport (COT), representing the energy consumption required for robot movement, was calculated, and a minimum COT of 0.08 was recorded, which is comparable to that of an actual fish.

## 1. Introduction

Underwater robots are becoming increasingly important in various fields, such as ecological research of marine organisms, inspection and investigation of underwater facilities, and search and rescue [1].

Currently, the majority of underwater robots are propelled by screws. However, underwater screw robots face the problems of difficulty in achieving both a good propulsion and turning performance, as well as negative effects on the environment due to entrainment and noise caused by high-speed screw rotation.

As a solution to these problems, biomimetic fish robots, which focus on the swimming methods of living organisms, are receiving attention [2]. In the early stages of the development of fish robots, multi-link fish robots were developed by connecting multiple motors and rigid links [3,4], and motor-crank fish robots were developed by using a crank mechanism to move one or more links by rotation of a single motor [5,6]. While these robots can produce the target motion with high precision, multi-link robots have a complex structure and tend to be bulky, and motor-crank robots cannot perform motions other than the pre-designed crank motion.

In recent years, the research and development of fish robots based on soft robotics, which incorporates soft materials and soft actuators, has been attempted. Soft material robots include robots with a motor and a soft body [7], robots with a motor/wire mechanism and a soft body [8,9,10], and robots with a tensegrity mechanism that allows the rigidity of the body to be set to an arbitrary value at each body length position [11,12]. Meanwhile, robots have been developed using soft actuators such as pneumatic and hydrodynamic actuators that deform by fluid pressure [13,14]; shape memory alloys (SMA) that shorten by heating [15,16,17,18]; and piezoelectric composite fiber [19], dielectric elastomer actuators (DEA) [20,21], and hydraulically amplified self-healing electrostatic (HASEL) actuators [22] that deform by applying a voltage.

The soft parts of these robots perform multiple motions, making it possible to develop fish robots that are more compact and capable of performing multiple swims, unlike rigid robots. On the other hand, it is difficult to generate a variety of motions with high precision because the motion of the soft parts is greatly affected by the stiffness and the fluid force.

We focus on the anatomical structure of underwater organisms to solve the problem of the difficulty in controlling soft robots with high precision. In the first step, a simple robot that mimics the structure of red muscle was fabricated using shape memory alloy wire as an actuator; the basic feasibility of the mechanism has been shown in our previous work [23].

In this paper, an improved structural design method considering the structure of the robot and a method for designing the input considering the characteristics of the shape memory alloy were studied in order to improve the performance of the robot. In addition, the swimming performance of the robot was evaluated using several evaluation items. The robot was named UEC Mackerel.

The rest of the paper is organized as follows: in Section 2, the mechanism of fish swimming is explained; in Section 3, the structure of fish red muscle is explained; in Section 4, the design of the UEC Mackerel is explained; in Section 5, an improved structural design method and method for designing the input are explained as well as the result of the experiment; and finally, Section 6 is dedicated to the discussion and the conclusion.

## 2. Mechanism of Fish Swimming

### 2.1. Modes of Fish Swimming

Fish swimming can be generally classified into the following two types depending on the part of the body mainly used for propulsion [24]. The body and/or caudal fin (BCF) swimming mode generates hydrodynamic force by moving the body and caudal fins to the sides and is superior in propulsion performance, while the median and/or pectoral fin (MPF) swimming mode generates hydrodynamic force by moving the pectoral and dorsal fins, enabling fine attitude control and turning performance. BCF swimming modes are further classified as auguilliform, subcarangiform, carangiform, thunniform, and ostraciiform, depending on whether their motion is undulatory or oscillatory.

In the Auguilliform swimming mode, the entire body is significantly deformed at low swimming frequency to generate undulation motion. On the other hand, in the subcarangiform, carangiform, and thunniform swimming modes, the swimming motion is performed by undulation motion using the posterior part of the body and caudal fin, and the more oscillations, the higher the swimming frequency and the smaller the deformation. The undulatory mode is superior in turning performance, while the oscillatory mode is superior in propulsive performance. The purpose of this paper is to develop a compact robot with high propulsive performance; therefore, the details of the carangiform swimming mode are investigated, characteristic of fish with a compact size and oscillatory motion.

### 2.2. Undulation and Input Signal

The relationship between the undulation motion and the input signal in fish in steady swimming has been reviewed by Shadwick et al. [25].

The undulation motion is generated by the contraction of the muscles on both sides of the body. The left and right muscles at the same body length position contract alternately, and the longitudinally aligned muscles on one side contract sequentially backwards. Muscle contraction generates a bending moment in the vertebrae that causes the body to bend, and generates undulation motion with increasing amplitude of lateral displacement to the posterior.

The amplitude of the lateral displacement at each part of the body is constant in most fish regardless of swimming speed and frequency, and the amplitude in the caudal fin is constant at about 0.2BL, where BL is the total body length.

The signal to the muscle shows the same behavior as the timing of muscle contraction. There is a phase difference of 180 deg in the signals to the left and right muscles at the same body length position, and there is a delay towards the back of the body. The velocity of the undulation motion and the phase difference between the head and the caudal fin of various fish are shown in Table 1. The values refer to reference [25]. As shown in Table 1, the phase difference of a mackerel, which is known to use the carangiform swimming mode, is about 225–300 deg.

### 2.3. Muscle Strain and Inter-Vertebral Bending

Muscle contraction and body curvature also increase backwards, as predicted by the posteriorly increasing lateral amplitude of undulation motion. The muscle strain in mackerels was measured by sonomicrometry and calculated from body bending. The muscle strain of mackerels was about ±4% at 0.3BL and increased linearly posteriorly, reaching a maximum of about ±8% at 0.8BL. On the other hand, the curvature and lateral amplitude of undulation motion increased quadratically backwards [26].

Based on the information above, the following two points were set as the design requirements of the robot:

1. Generate undulation motion at various phase differences.
2. The inter-vertebral bending angle must increase quadratically backwards.

### 2.4. Swimming Performance of Fish

The distance traveled forward in one tail beat cycle is the stride length, $\lambda_{s}$, and is expressed using the swimming speed, *u*, the period of tail beat, *T*, and the tail beat frequency, *f*, as:

(1) $$\begin{array}{c} \lambda_{s}=\left(u/BL\right)\cdot T=\left(u/BL\right)/f. \end{array}$$

The stride lengths of subcarangiform, carangiform, and thunniform swimmers are 0.60–0.70, while the stride lengths of Auguilliform swimmers are between 0.35 and 0.50.

The propulsion efficiency (Froude efficiency) is the ratio of the propulsive force to the total power and is expressed as follows [27]:

(2) $$\begin{array}{c} \eta_{f}=\frac{u+v}{2v}=\frac{u}{2v}+\frac{1}{2}=\frac{\lambda_{s}}{2\lambda_{b}}+\frac{1}{2}, \end{array}$$

where $\lambda_{b}$ represents the wavelength of undulation motion and is expressed as 360/ϕ[BL]. From Equation (2), the closer *u* is to *v*, the less energy is consumed by lateral motion and the more optimal swimming becomes.

Both the stride length and propulsive efficiency are used to evaluate the robots in this paper.

## 3. Structure of Fish Red Muscle

In mackerel, muscle fibers are classified into two types, red, slowly contracting aerobic muscles and white, fast-contracting anaerobic muscles [28,29]. In this paper, the red muscle used for steady-state swimming is investigated.

The red muscle of mackerel is positioned just behind the skin at the vertebral height of the horizontal plane. In the same plane, a layer of connective tissue called the main horizontal septum (MHS) is present, which is attached to the vertebrae. The MHS consists of a lattice of two types of tendons, the anterior oblique tendons (AOTs) and the posterior oblique tendons (POTs). In the MHS, AOTs extend from the vertebrae posteriorly outward to the skin, while POTs extend from the vertebrae anteriorly to near the skin. The corresponding AOT and POT overlap at a position near the skin, forming an AOT–POT loop.

**Figure 1:**
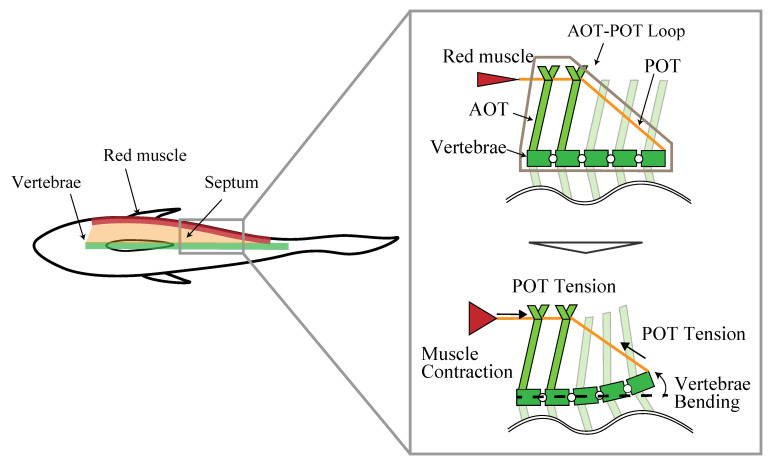
Model of AOT–POT loop and red muscle.

Westneat et al. proposed a model in which red muscle contraction is transmitted to the vertebrae by this AOT–POT loop [28]. The model is shown in Figure 1. In this model, the POT connects the red muscle to the vertebrae and acts as a wire, and the contraction force of the red muscle is transmitted to the vertebrae as tension by the POT. The AOT plays the role of a beam that holds the red muscle and part of the POT on the outside of the body.

The characteristic values of the AOT–POT loop of mackerel are shown in Table 2. The values refer to reference [28]. The incident angle of AOT is about 45–55 deg in the front and increases backward, eventually reaching 75–80 deg. The incident angle of POT is about 45 deg in the front, and decreases backward, eventually reaching about 20 deg. The number of vertebrae included in the AOT–POT loop is five in the anterior region and decreases toward the posterior region, eventually reaching three.

## 4. Design of UEC Mackerel

### 4.1. Design of Bending Module

A bending module that can be connected in the longitudinal direction was designed by focusing on the AOT–POT loop described above. In this paper, the conversion mechanism between muscle contraction and vertebral bending was studied. The concept of the drive module is described below.

1. Each module is independent of the other and each mimics a single AOT–POT loop.
2. The vertebrae in the AOT–POT loop approximate a single rigid link.
3. To obtain sufficient strain, both the red muscle and POT were replaced with soft actuators (a shape memory alloy (SMA) wire).
4. The tip of the actuator is connected to the AOT beam and the trailing edge of the next link.

**Figure 2:**
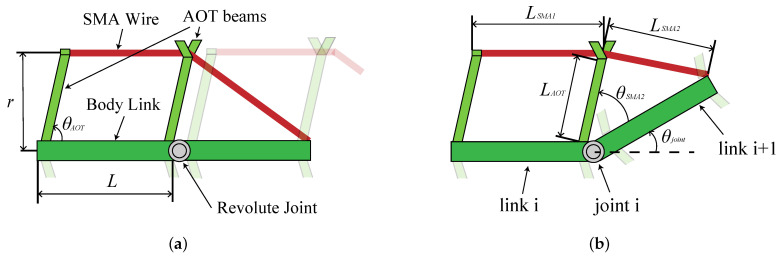
The designed bending module. (**a**) Modules not in operation. (**b**) Bending motion of the module.

The designed bending module and the bending motion during the drive are shown in Figure 2. As a result of the complexity and difficulty of incorporating the anatomical structure of the fish directly, modules were simplified as a first step toward developing a robot that mimics a fish swimming with high precision. Modules checked whether the design requirements could be met with the simplifications.

By connecting such modules in the longitudinal direction and driving each of them independently, it is considered possible to generate undulation motion with various phase differences, thus satisfying the design requirement 1 listed in Section 2.3.

Next, to verify whether design requirement 2 listed in Section 2.3 can be satisfied, the design parameters (length of body link, *L*, body width, *r*, and angle of AOT beam, $\theta_{AOT}$) were set and their effects on bending were observed.

The bending curvature, κ, when the actuator generates a strain of ϵ as shown in Figure 2b is calculated geometrically by following the steps. $L_{sma1}$, $L_{sma2}$, and $L_{AOT}$ are the lengths of SMA wire parallel to the body link, SMA wire oblique to the body link when bending motion is generated, and the rigid beam, respectively, and are expressed by the following:

(3) $$\begin{array}{cc} \; & L_{SMA1}=L\\ \; & L_{SMA2}=\left(L_{SMAdef}-L_{SMA1}\right)-\epsilon \cdot L_{SMAdef}\\ \; & L_{AOT}=\frac{r}{sin(\theta_{AOT})}, \end{array}$$

where $L_{SMAdef}$ represents the total length of the SMA when bending motion is not generated and is calculated as follows:

(4) $$\begin{array}{c} L_{SMAdef}=\sqrt{{L_{AOT}}^{2}+L^{2}-2\cdot L_{AOT}\cdot L\cdot cos\left(\theta_{AOT}\right)}+L_{SMA1}. \end{array}$$

Then, the bending curvature, κ, is expressed as follows:

(5) $$\begin{array}{cc} \; & \theta_{SMA2}=Cos^{-1}\left(\frac{L_{AOT}^{2}+L^{2}-L_{SMA}^{2}}{2\cdot L_{AOT}\cdot L}\right)\\ \; & \theta_{joint}=\theta_{AOT}-\theta_{SMA2}\\ \; & \kappa =\theta_{joint}/L. \end{array}$$

**Figure 3:**
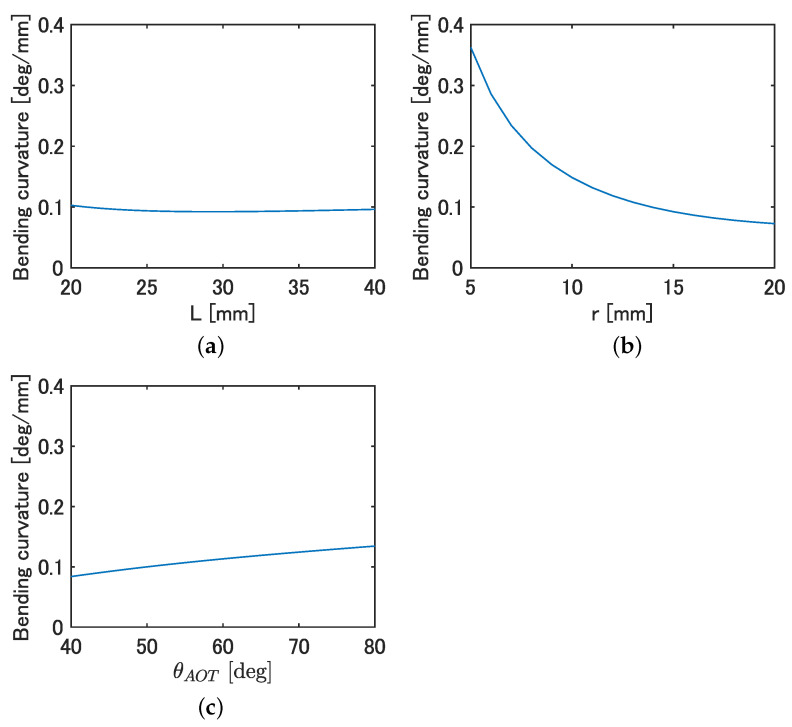
Effect of each parameter on curvature. (**a**) Curvature at each *L*. (**b**) Curvature at each *r*. (**c**) Curvature at each $\theta_{AOT}$.

The bending curvature was calculated by fixing two of the parameters of *L*, *r*, and $\theta_{AOT}$ and varying one using Equations (3)–(5). Values of *L* = 30 mm, *r* = 15 mm, and $\theta_{AOT}$ = 60 deg were used when each design parameter was fixed, and ϵ=±0.02 was used regardless of the conditions. Figure 3a–c shows the curvature when *L*, *r*, and $\theta_{AOT}$ were varied, respectively.

From Figure 3, a minimal effect on the curvature with changing *L* was observed, while a significant decrease in the curvature with increasing *r* and a slight increase in curvature with increasing $\theta_{AOT}$ was observed. Since the *r* of the fish decreases due to the tapered shape of the body and the observed $\theta_{AOT}$ increases backwards, as shown in Table 2, it is likely that fish tend to bend more backwards. Based on the above, there is a possibility that design requirement 2 is satisfied by connecting bending modules that mimic the parameters at each body length position of the fish.

### 4.2. Design of Fish Robot

**Figure 4:**
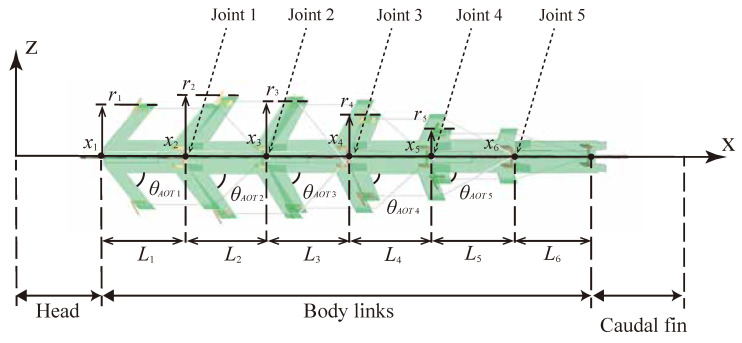
Designed UEC Mackerel.

The robot was designed by connecting six bending modules and it was termed UEC Mackerel. The prototype UEC Mackerel designed based on the design parameters is shown in Figure 4, and the values of the design parameters for each module, which were set to mimic the anatomy of a fish, are shown in Table 3. Methods for setting the values of each design parameter are described below.

First, *L* was set to 30 mm for all modules because the effect of the length on the bending curvature was considered to be small. As each link represents five vertebrae, six modules correspond to a total of thirty vertebrae, which is close to the actual number of mackerel vertebrae. In addition, it is assumed that the head and caudal fin parts of the same length as the body are connected to the front and back of the body.

Next, *r* was set based on the mathematical model of the mackerel body shape designed by Alvarado [7]. In Alvarado’s model, the body width, $r_{i}$, is calculated by the following:

(6) $$\begin{array}{cc} \; & r_{i}=c_{1}sin\;\left(c_{2}x_{i}\right)+c_{3}sin\;\left(c_{4}x_{i}\right), \end{array}$$

while the body height, $R_{i}$, is calculated by the following:

(7) $$\begin{array}{cc} \; & R_{i}=C_{1}sin\;\left(C_{2}x_{i}\right)+C_{3}\left(e^{C_{4}x_{i}}-1\right), \end{array}$$

where $x_{i}$ is the position in the body length direction. The head and caudal fin parts are attached to the front and back of the body with the same length as the links; therefore, the value of $x_{i}$ starts at 30 mm and ends at 180 mm. The body width, $r_{i}$, and height, $R_{i}$, at each body length position can be obtained by substituting the values in Table 4 into Equations (6) and (7). Values in Table 4 are taken from reference [7].

Finally, $\theta_{AOT}$ was set to increase linearly from 45 deg to 80 deg, referring to Table 2.

**Figure 5:**
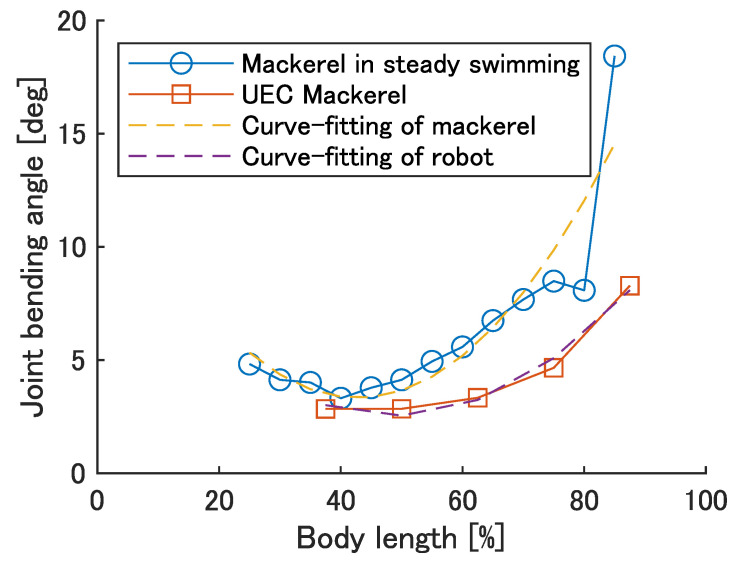
The maximum bending angle of the robot and fish.

The bending angle of modules when the soft actuator is under the strain of ϵ=±0.02 was calculated using the same method as in Section 4.1. The results are shown in Figure 5. Mackerel bending angles were calculated from the inter-vertebral bending angles in reference [26]. The yellow and purple dashed lines are the result of curve fitting the quadratic functions of the mackerel and robot bends, respectively, and are represented by the equations $y=0.0063x^{2}-0.5395x+14.8782$ and $y=0.0037x^{2}-0.3601x+11.3172$, respectively. Figure 5 shows a quadratic increase backwards, suggesting that design requirement 2 is satisfied, although it is small compared to the bending angle of the fish.

**Figure 6:**
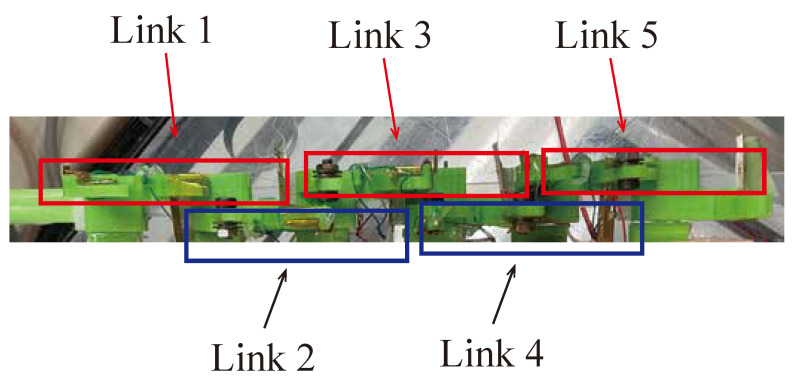
View of the robot from the side.

When the modules are arranged in the same plane, the front and back AOT beams come into contact with each other when bending. Therefore, the height of adjacent wire structures is divided as shown in Figure 6.

### 4.3. Shape Memory Alloy Wire Actuator Used for the Robot

In this paper, shape memory alloy (SMA) wires were used as actuators. SMA has the property of recovering strain induced by the loading process when heated based on a thermo-elastic martensitic transformation [30]. By applying a voltage to both ends of the SMA, it can be heated and contracted by resistive heat. In this paper, Toki Corporation’s BMF75 SMA was chosen because it requires a lower voltage and produces strain similar to that of red muscle [31] (refer to Table 5).

**Figure 7:**
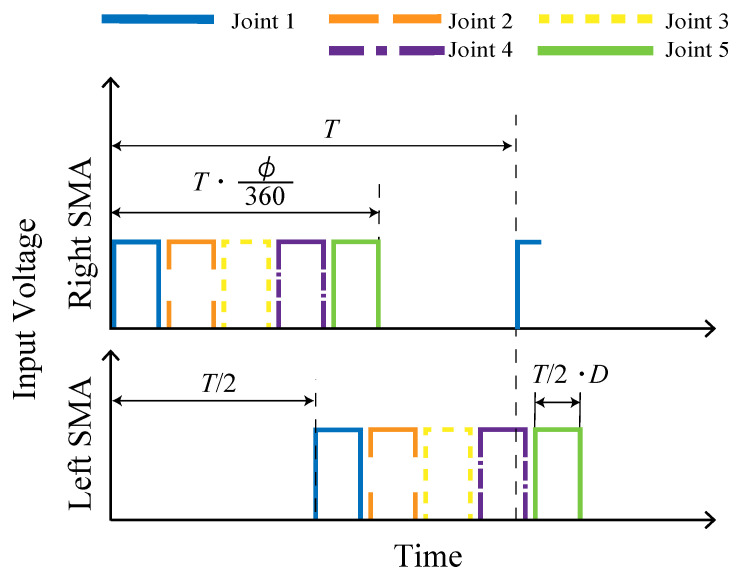
Input to the SMA.

Figure 7 shows the voltage input to the left and right SMAs of each module. When the period *T* (s) and phase difference ϕ (deg) are set, the elapsed time from the start time of application to the first module to the end time of application to the fifth module is expressed as T·ϕ/360 (s). In addition, when the duty ratio of each input is *D*, each voltage is applied for T/2·D (s).

**Figure 8:**
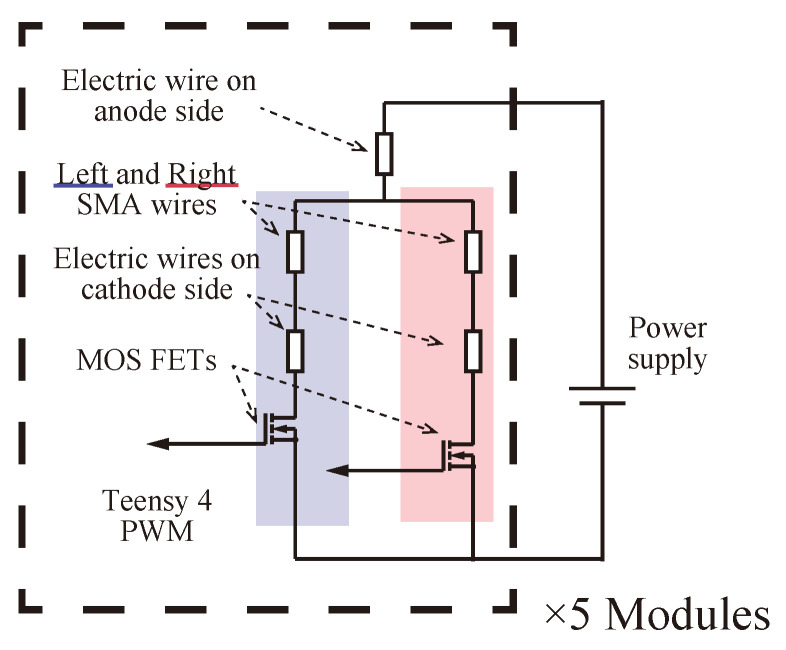
The electrical model of the module control unit.

Figure 8 shows the electrical model of the module control unit. Each SMA wire is driven by applying a signal voltage from the microcomputer Teensy 4.0 to the MOSFETs at the time shown in Figure 7.

By providing such an input, undulation motion is expected to be generated by delayed backwards bending, thereby satisfying design requirement 1.

## 5. Improved UEC Mackerel

### 5.1. Design of Improved UEC Mackerel

In our previous work, the prototype of the robot with a similar length of SMA wire in each module was developed, focusing on the fact that the amount of red muscle in mackerel is constant at the majority of longitudinal positions [23]. Experiments in air and insulating fluid were conducted to evaluate the robot performance. The experiments in air confirmed that undulation motion of various phase differences is generated by controlling the input phase difference, ϕ, and that the maximum bending angle of each joint exhibits a quadratic trend, therefore satisfying design requirements 1 and 2. Experiments in insulating fluid confirmed that the robot was able to swim, but its performance was poor under all conditions except when ϕ=120 deg. The main cause of this problem was considered to be the similar length of the SMA wire in each module. However, the amount of contraction strain in the red muscle of a mackerel increases linearly from 4% in the front to about 8% near the caudal peduncle, whereas the SMA wire used in this paper does not have a contraction strain of more than 4% due to its characteristics. As a result, the maximum bending angle of the bending module could not reproduce the value of the mackerel, especially in the backward direction. Considering this to be the cause of the decrease in swimming speed, the length of the SMA wire was increased toward the caudal region, using pulleys to mimic the muscle strain of fish.

**Figure 9:**
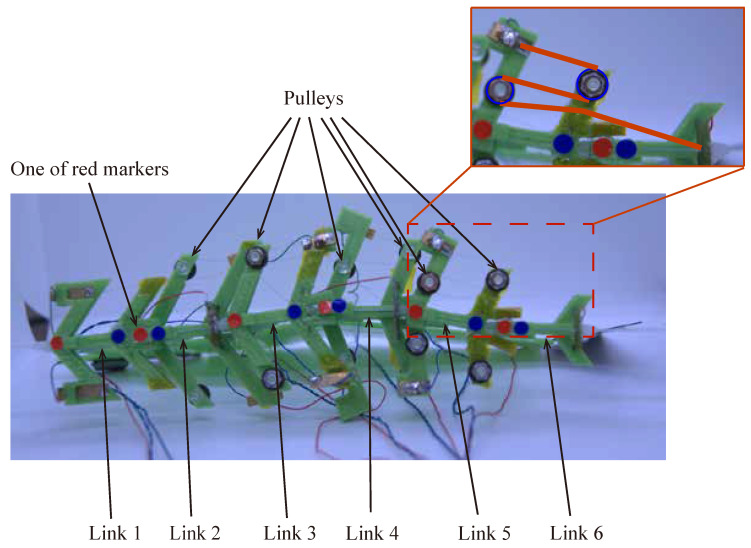
Improved UEC Mackerel from above. In the detailed view, the blue circles represent the pulley, and the red lines represent the path of the SMA wire.

Figure 9 shows the improved UEC Mackerel design. As shown in Figure 9, the SMA wire is reciprocated using a pulley to increase the length of the SMA wire without changing the design parameters, thereby increasing the extent of contraction. The first module has no reciprocation, the second and third modules have one reciprocation, and the fourth and fifth modules have two reciprocations.

The length of the SMA wire in each module and the value corresponding to the strain on the muscle, $s_{muscle}$, are shown in Table 6. $s_{muscle}$ was calculated using $s_{muscle}=0.02\cdot L_{SMA}/30$, assuming that the virtual red muscle length is 30 mm, the same value as the length of body links.

### 5.2. Input for Improved UEC Mackerel

The optimal voltage and application time for the improved UEC mackerel were reexamined. The thermal model of the SMA wire is expressed by the following [30]:

(8) $$\begin{array}{c} m_{sma}c_{p}\frac{d\Theta }{dt}=I_{sma}^{2}R_{sma}-h_{c}A_{c}\left(\Theta -\Theta_{\infty }\right), \end{array}$$

where $m_{sma}$, $c_{p}$, Θ, *t*, $I_{sma}$, $R_{sma}$, $h_{c}$, $A_{c}$, and $\Theta_{\infty }$ represent the mass per unit length of SMA wire, the specific heat of the wire, the temperature of the SMA wire, the time, the current applied to the SMA wire, the resistance per unit length of the SMA wire, the thermal conductivity of the SMA wire, the lateral area per unit length of the SMA wire, and the room temperature, respectively. By integrating both sides of Equation (8), the time required to reach temperature Θ when a constant current $I_{sma}$ is applied at room temperature $\Theta_{\infty }$ is obtained as follows:

(9) $$\begin{array}{c} t=-log\left|-\frac{h_{c}A_{c}}{m_{sma}c_{p}}\left(\Theta -\Theta_{\infty }\right)+\frac{I_{sma}^{2}R_{sma}}{m_{sma}c_{p}}\right|+C, \end{array}$$

where *C* is the integration constant.

Consider starting at room temperature $\Theta_{\infty }$ and heating up the SMA wire to a temperature $A_{f}$ where phase transformation occurs at a constant voltage $V=I_{sma}R_{sma}$. $C=log\left|I_{sma}^{2}R_{sma}/m_{sma}c_{p}\right|$ is obtained by substituting the initial conditions t=0 and $\Theta =\Theta_{\infty }$ into Equation (9). Substituting *C* and $\Theta =A_{f}$ into Equation (9), the time, $t_{Af}$, required to heat up to $A_{f}$ at a constant voltage *V* is obtained as follows:

(10) $$\begin{array}{c} t_{Af}=log\left|\frac{V^{2}}{V^{2}-a}\right|, \end{array}$$

where $a=R_{sma}h_{c}A_{c}\left(A_{f}-\Theta_{\infty }\right)$.

Meanwhile, when cooling the SMA wire from $A_{f}$ to $\Theta_{\infty }$ without applying voltage, $C=log\left|h_{c}A_{c}/m_{sma}c_{p}\right|\cdot \left(A_{f}-\Theta_{\infty }\right)$ is obtained by substituting the initial conditions t=0, $\Theta =A_{f}$, and $I_{sma}$ = 0 into Equation (9). Substituting *C* into Equation (9), the time, $t_{Mf}$, required for the SMA wire to cool from $A_{f}$ to room temperature when no voltage is applied to the wire is expressed as follows:

(11) $$\begin{array}{c} t_{Mf}=log\left|\frac{h_{c}A_{c}}{m_{sma}c_{p}}\left(A_{f}-\Theta_{\infty }\right)\right|=b. \end{array}$$

Since the parameters in Equation (11) are the properties of the SMA wire and the room temperature can be assumed to be constant, $t_{Mf}$ takes a constant value *b*.

**Figure 10:**
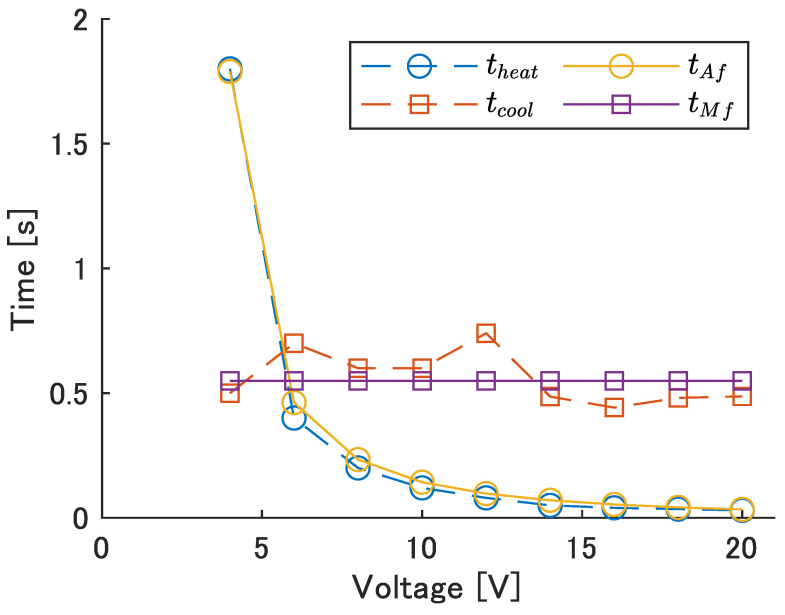
The time required to heat and cool.

The fifth module of UEC Mackerel was used to apply various voltages to the SMA wire on one side and measure the time until the maximum bending angle appeared, $t_{heat}$. The time taken to return to the original position when the voltage was stopped, $t_{cool}$, was also measured, and *a* and *b* from Equations (10) and (11) were obtained using the least squares method. The experimental results and the obtained model are shown in Figure 10. The values of the model are a=13.3 and b=0.55.

The sum of $t_{Af}$ and $t_{Mf}$ is the time it takes for the module to return to its original position after it begins to bend to one side, and is related to the period *T* and frequency *f* of left–right motion by:

(12) $$\begin{array}{c} T=1/f=2(t_{Af}+t_{Mf}). \end{array}$$

Table 7 shows the voltage, $V_{input}$, voltage application time, $t_{Af}$, and calculated average power consumption per cycle, $\bar{P}$, required to bend to the left and right at each frequency. The input time, $t_{Af}$, for each input frequency is calculated by substituting the frequency and $t_{Mf}$ = 0.55 s into Equation (12). Next, the voltage required to heat the SMA wire to temperature $A_{f}$ in the input time $t_{Af}$ is calculated by substituting the obtained $t_{Af}$ into Equation (10). $\bar{P}$ was calculated from $\bar{P}=V_{input}^{2}/R_{sma}\cdot t_{Af}/T$. The value of $R_{sma}=27$ Ω, corresponding to the resistance of a 110 mm SMA wire, was used for the calculation. The maximum input frequency was set at 0.8 Hz because a large increase in voltage occurred at 0.9 Hz, as seen in Table 7.

### 5.3. Experiment Using the Improved UEC Mackerel

**Figure 11:**
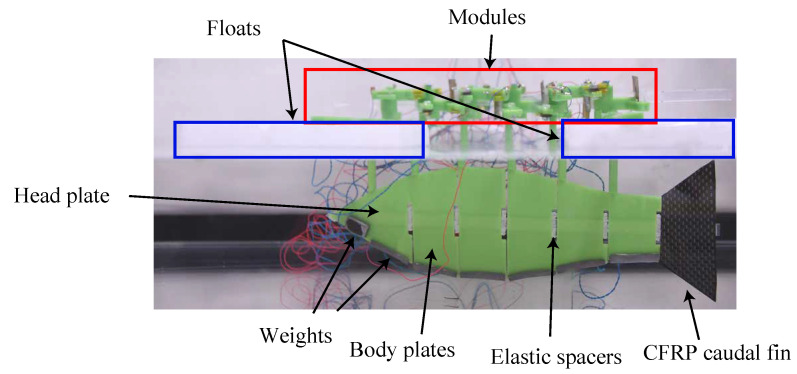
Improved UEC Mackerel for swimming experiment.

A fish-shaped flat plate was attached to the bottom of the robot, floats were placed on the first and last module, and a 10 g weight was placed on each module to prevent the modules from touching the insulating fluid. The module floats on the fluid surface to avoid the SMA wire being cooled by the fluid and shortening. It also serves to prevent short circuits in future experiments using water instead of insulating fluid. On the other hand, because the module and the fish-shaped plate do not exist on the same surface, the module undergoes oscillations not associated with the fish undulation motion. Therefore, the module will be submerged after waterproofing with soft skin in future work. The shape of the fish-shaped plate was designed using $R_{i}$ from Equation (7). The assembled robot is shown in Figure 11.

Fluorinert FC-3283 manufactured by 3M was used as the insulating fluid [32]. FC-3283 has a density of 1830 kg/m${}^{3}$ and a kinematic viscosity of 0.75 cSt. Compared to water (at 25 °C) with a density of 997 kg/m${}^{3}$ and 0.89 cSt, the density is about twice as high, while the kinematic viscosity is about 0.84 times higher, roughly equal to water.

Swimming experiments were conducted in an insulating fluid on the improved robot in the same manner as on the prototype robot. In addition to swimming speed, the stride length, propulsion efficiency, swimming shape, and cost of transport (COT) were evaluated.

#### 5.3.1. Swimming Speed and Stride Length of Improved UEC Mackerel

Current robots are not able to swim straight due to biased undulation motion to the left and right in each period T, especially at low phase differences, and biased left–right motion due to manufacturing errors. Therefore, the swimming speed of the robot was measured using the following procedure:

1. A video is taken when the robot crosses about 200 mm directly under the camera.
2. The coordinates of the marker in front of link 1 are tracked.
3. The direction in which the marker’s position is passed at the beginning and the end of the video is set as the direction of propulsion.
4. The distance, δx, traveled in the direction of propulsion every 0.167 s was measured and divided by 0.167 s to calculate the swimming speed at each time.
5. The average of the swimming speed at each time is used as the swimming speed of the robot.

**Figure 12:**
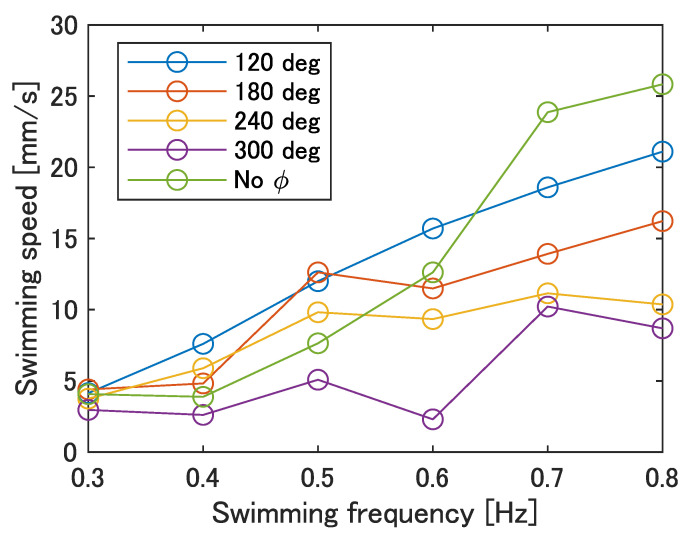
Swimming speed of the improved UEC Mackerel.

Figure 12 shows the swimming speed at each swimming frequency. The swimming frequency was assumed to be the same value as the input frequency. As shown in Figure 12, the maximum swimming velocity was 25.8 mm/s at a frequency of 0.8 Hz with no phase difference. Furthermore, compared to the prototype in our previous work [23], the swimming speed tended to increase with an increase in frequency at all phase differences. Therefore, an improvement in the swimming performance can be expected at any phase difference as the robot’s frequency response improves.

**Figure 13:**
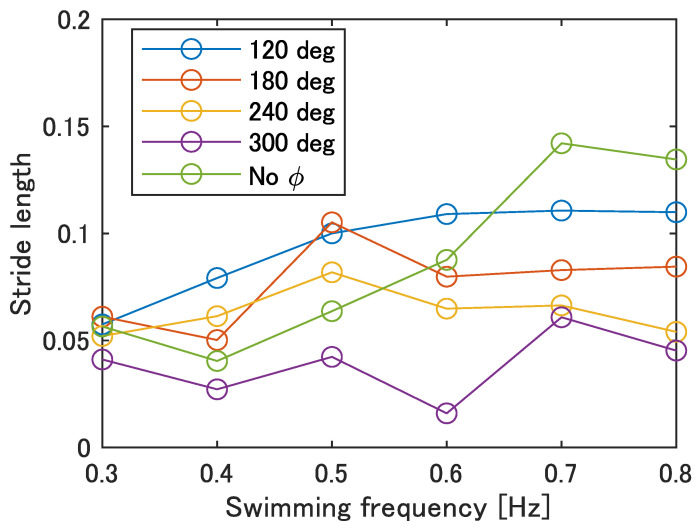
Stride length of the improved UEC Mackerel.

Figure 13 shows the stride length at each swimming frequency calculated by Equation (1). In Figure 13, the maximum stride length was 0.14 at 0.7 Hz with no phase difference, and the minimum was 0.02 at 0.6 Hz with ϕ = 300 deg. Compared to actual fish, the stride length of the prototype fish is about 40% of that of auguilliform swimmers and 23% of that of subcarangiform and thunniform swimmers, indicating that the stride length needs to increase by improving the swimming speed.

The swimming speed and stride lengths of the fish robots developed in the paper and by other studies are shown in Table 8. As shown in Table 8, the improved UEC Mackerel achieved a comparable swimming speed or stride length compared to the other robots.

#### 5.3.2. Propulsion Efficiency and Swimming Shape of Improved UEC Mackerel

To determine the cause of the low swimming speed and stride length compared to the actual fish, the propulsive efficiency and swimming shape of the robot were observed and compared with those of the fish.

**Figure 14:**
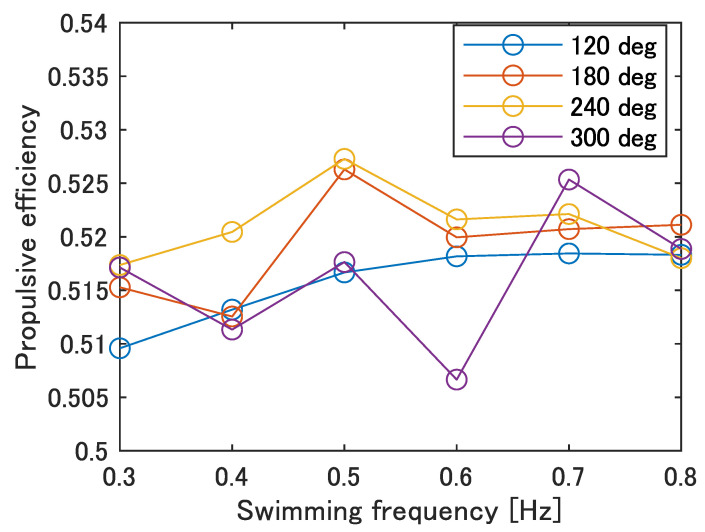
Propulsive efficiency of improved UEC Mackerel.

Figure 14 shows the propulsive efficiency at each swimming frequency calculated by Equation (2). As shown in Figure 14, ϕ = 240 deg was the maximum at the most of the frequencies, especially at 0.5 Hz with an overall maximum value of 0.527. The phase difference of the carangiform swimmer is about 225–300 deg, which is consistent with the trend of the robot. Therefore, it is possible that the parameters of no phase difference and ϕ = 120 deg, which currently result in the highest swimming speed, do not allow for efficient swimming, and improvement in the swimming performance at high phase differences is required.

**Figure 15:**
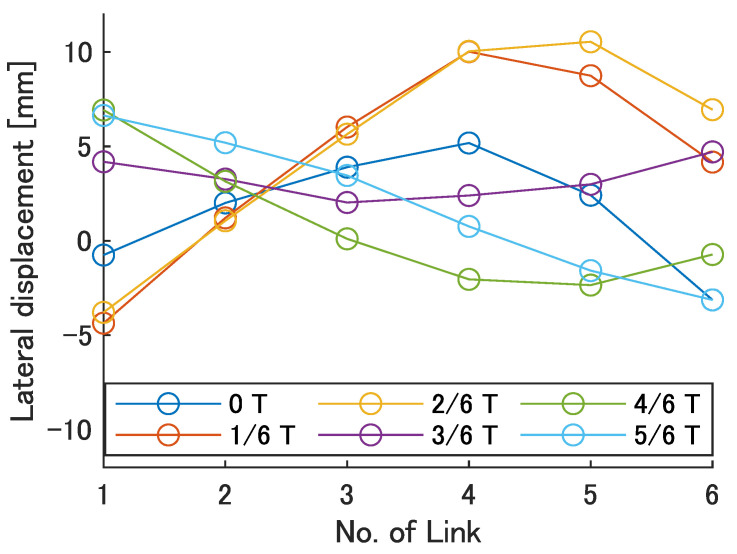
Swimming shape with no phase difference.

**Figure 16:**
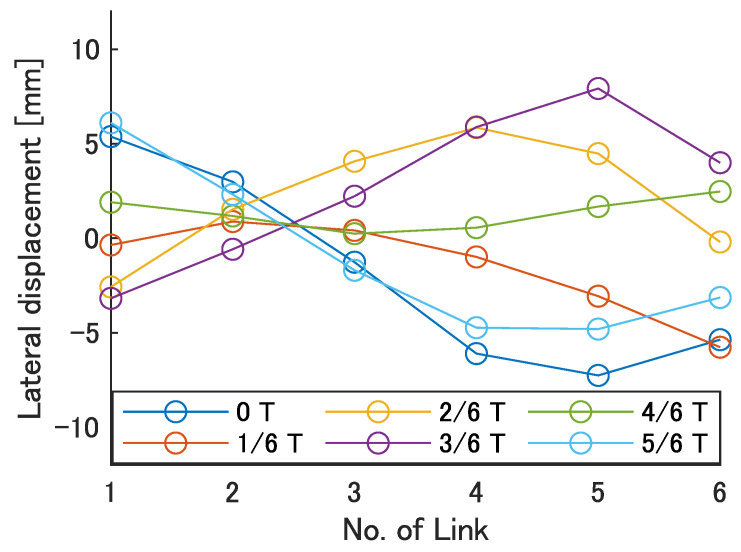
Swimming shape when ϕ = 120 deg.

**Figure 17:**
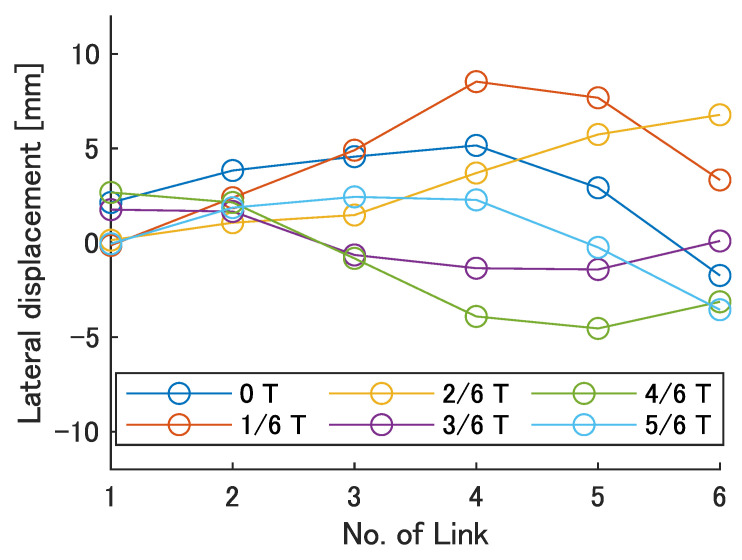
Swimming shape when ϕ = 180 deg.

**Figure 18:**
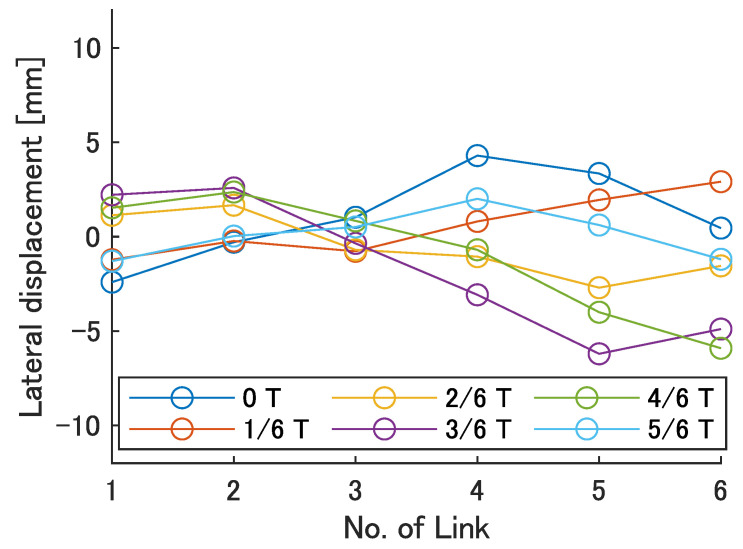
Swimming shape when ϕ = 240 deg.

**Figure 19:**
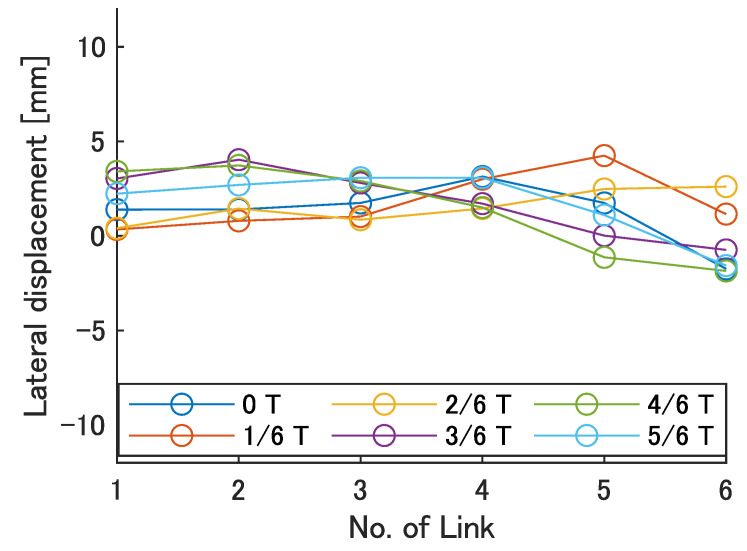
Swimming shape when ϕ = 300 deg.

Next, the swimming shape was examined by measuring the lateral displacement of the red marker on each link, as shown in Figure 9. Figure 15, Figure 16, Figure 17, Figure 18 and Figure 19 show the lateral displacement of each link in one cycle when the swimming frequency is 0.8 Hz and the phase difference is ϕ = 120–300 deg or there is no phase difference.

Focusing on the position of the peak of the lateral amplitude at each ϕ of the swimming shape, the peak was observed at the last link only when there was no phase difference and ϕ = 120 deg, which indicated a high swimming velocity. This suggests that in order to improve the swimming performance at high phase differences, the peak of lateral amplitude should be located near the caudal fin during high phase difference swimming.

In terms of the magnitude of the amplitude of lateral displacement at the caudal region, the maximum value in the experiment was 7.5 mm at ϕ = 120 deg, while the value for the fish is about 48 mm, which is 0.2 times the robot’s total length. Therefore, the decrease in swimming performance due to the small tail fin size is also considered to be significant.

#### 5.3.3. Cost of Transport of the Improved UEC Mackerel

**Figure 20:**
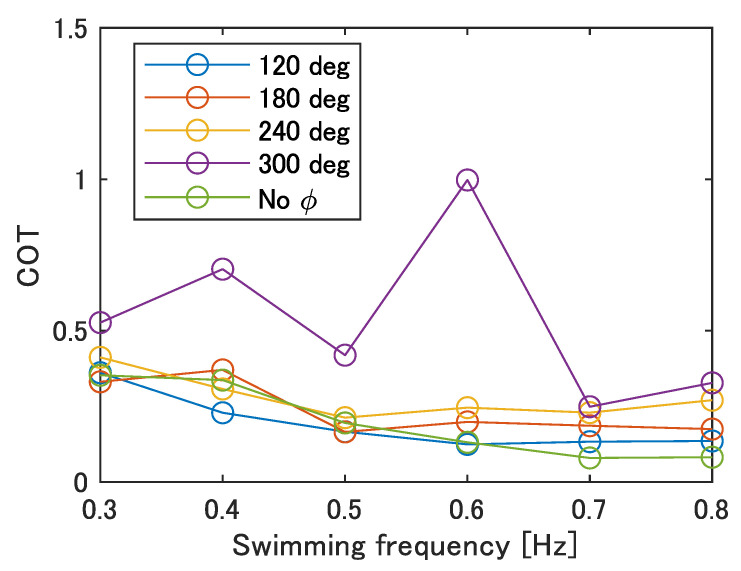
COT of the improved UEC Mackerel.

Finally, the cost of transport (COT) was evaluated to examine the robot’s mobility performance. The COT is obtained by the following:

(13) $$\begin{array}{c} COT=\frac{P}{mgu} \end{array}$$

where *P*, *m*, *g*, and *u* are the power consumption, the mass of the robot, the gravitational acceleration, and the swimming speed, respectively. The power consumption was obtained from the multiplication of the voltage and the current of the power supply. The result is shown in Figure 20. The minimum value of the COT is 0.08 when there is no phase difference at 0.7–0.8 Hz, and the maximum value of the COT is 1.00 when ϕ = 300 deg at 0.6 Hz.

The reasons for the generally high COT at ϕ = 300 deg, with a maximum value at 0.6 Hz, are considered to be as follows. Focusing on Equation (13), the two variables are the swimming speed, *u*, and the power consumption, *P*. The price of the COT increases when the swimming speed, *u*, decreases and the power consumption, *P*, increases. Figure 12 shows that the velocity of movement, *u*, takes on smaller values for lower frequencies and for larger phase differences. In contrast, Table 7 shows that the calculated average power consumption, $\bar{P}$, per cycle is larger at higher frequencies. Therefore, the COT was high at ϕ = 300 deg, where the swimming velocity was overall low, and the maximum COT was observed at 0.6 Hz, where the swimming speed was low despite the increase in frequency. On the other hand, at 0.7–0.8 Hz, where the COT reaches its minimum value for each ϕ, the smaller the value of ϕ, the smaller the COT and the more efficient the swimming. Since the power consumption is the same regardless of the phase difference at the same frequency, the cause is considered to be the decrease in the swimming speed.

The COT of carp observed in other research was about 0.1–0.3 [33]. Thus, the robot moves with an energy efficiency close to that of fish in swimming motion, except for at ϕ = 300 deg. However, since the power supply and electric circuits are located outside of the robot, it is necessary to pay attention to the effect of incorporating these into the robot on the COT in the future.

## 6. Discussion and Conclusions

In this paper, we developed an improved bending module that mimics the red muscle and its related tendons and vertebrae in order to reproduce the efficient, steady swimming of fish, which is highly sustainable. We also developed an improved underwater robot by connecting the bending module and observed its motion in insulating fluid.

The improved robot using pulleys was designed and fabricated because the small extent of SMA contraction in the back was considered to be problematic. The swimming speed increased to a maximum value of 25.8 mm/s at ϕ = 120 deg and 0.8 Hz. In addition, an increase in the swimming velocity with increasing frequency was observed for all values of ϕ. The stride length was also measured and the highest value of 0.14 was obtained at ϕ = 120 deg and 0.7 Hz. The swimming speed and stride length of the improved UEC Mackerel were confirmed to reach the same level of performance of soft robots in other studies, while they were inferior to those of actual fish.

To investigate the cause of this issue, the propulsive efficiency and swimming shape were observed, and the following results were found. The observations of propulsive efficiency showed a maximum value for ϕ of 240 deg at most input frequencies, which did not result in a high swimming speed. Observations of the swimming shape showed that the peak of lateral amplitude appeared near the tail fin in the two phase difference conditions that exhibited high swimming speeds, and that the lateral amplitude in the caudal region was smaller than that of an actual fish in all inputs. These results suggest that the swimming speed at high phase differences with high efficiency should be increased for better swimming performance, and in order to do so, the peak of lateral amplitude should be placed near the caudal fin and the value of the amplitude should be increased.

Finally, the COT was calculated to evaluate the mobility performance of the robot. Since the COT was less than 0.5 for all values of ϕ, except for ϕ = 300 deg, and since the COTs for fish are around 0.1–0.3, it can be assumed that the robot swims with a similar energy efficiency as fish.

In future work, we will try to reproduce the swimming performance of fish by improving the swimming speed at high phase differences. As one specific method to achieve this, the number of links in the AOT–POT loop will be increased, and the drive of multiple actuators will be transmitted to a single link to improve the smoothness of the undulation motion at high phase differences. We will also work on reproducing the gradual turning motion during steady swimming, which is likely to be generated by the red muscles.

## Figures and Tables

**Table 1:** Velocity and phase difference of the various fish.

	Undulation Velocity $v(\mathrm{BL}\cdot T^{-1})$	Phase Difference ϕ (deg)
Bass	1.3	277
Trout, Saithe, Mackerel, Carp, and Leopard Shark	1.2–1.6	225–300
Bonito, Mako Shark, Scup, and Yellow Fin Tuna	1.7–2.1	171–212
Eel and Lamprey	0.6	600

**Table 2:** Characteristic values of the AOT–POT loop of mackerel.

	Average	Front Edge	Back Edge
Body length	342.7 mm	-	-
Number of Vertebrae	31–32	-	-
Number of Vertebrae in AOT–POT loop	-	5	3
Incident angle of the AOT	-	45–55 deg	75–80 deg
Incident angle of the POT	-	45 deg	20 deg

**Table 3:** The values of the design parameters for each module.

Link No.	1	2	3	4	5	6
$x_{i}$ (mm)	30	60	90	120	150	180
$L_{i}$ (mm)	30	30	30	30	30	30
$r_{i}$ (mm)	17.2	20.1	18.5	13.7	8.0	-
$\theta_{AOTi}$ (deg)	50.7	56.5	62.4	68.3	74.1	-

**Table 4:** Parameters for the mathematical model of the mackerel body.

*j*	$c_{j}$	$C_{j}$
1	0.045 BL	0.14 BL
2	2π/1.25 BL	2π/1.6 BL
3	0.06 BL	0.0008 BL
4	2π/3.14 BL	2π/1.1 BL

**Table 5:** Specification of BMF75.

	BMF75
Standard diameter	0.075 mm
Practical strain	4.0%
Standard drive current	140 mA
Standard drive voltage	35.4 V/m
Tensile strength	0.45 Kgf
Weight	28 mg/m

**Table 6:** The length of SMA wire and the ratio of SMA wire contraction to the length of body link.

Link No.	$L_{\mathrm{SMA}}$ (mm)	$s_{\mathrm{muscle}}\;$(%)
1	49.4	3.3
2	76.7	5.1
3	78.5	5.2
4	109.3	7.3
5	110.1	7.3

**Table 7:** Input voltage and time at each input frequency.

Input Frequency [Hz]	$t_{\mathrm{Af}}$ (s)	$V_{\mathrm{input}}$ (V)	$\bar{P}$ (W)
0.3	1.1167	4.45	0.238
0.4	0.7000	5.15	0.267
0.5	0.4500	6.06	0.297
0.6	0.2833	7.35	0.330
0.7	0.1643	9.38	0.364
0.8	0.0750	13.58	0.400
0.9	0.0056	49.05	0.436

**Table 8:** Swimming performance of various fish robots.

	Swimming Speed (BL/s) (Frequency)	Stride Length
Improved UEC Mackerel	0.11 (0.8 Hz)	0.14 (0.7 Hz)
Prototype UEC Mackerel [23]	0.08 (1.3 Hz)	0.08 (0.7 Hz)
SMA fish [15]	0.76 (2.5 Hz)	0.30
SMA fish [16]	0.1 (0.5 Hz)	0.20
SMA fish [17]	0.46 (2.25 Hz)	0.20
SMA fish [18]	0.10 (0.5 Hz)	0.20
DEA fish [20]	∼0.07 (3 Hz)	0.023
DEA fish [21]	0.22 (6 Hz)	0.037
FEA fish [13]	0.44 (1.67 Hz)	0.26

## Data Availability

Not applicable.
